# The Establishment of Quantitatively Regulating Expression Cassette with sgRNA Targeting *BIRC5* to Elucidate the Synergistic Pathway of Survivin with P-Glycoprotein in Cancer Multi-Drug Resistance

**DOI:** 10.3389/fcell.2021.797005

**Published:** 2022-01-03

**Authors:** Changping Deng, Fabiao Hu, Zhangting Zhao, Yiwen Zhou, Yuping Liu, Tong Zhang, Shihui Li, Wenyun Zheng, Wenliang Zhang, Tianwen Wang, Xingyuan Ma

**Affiliations:** ^1^ State Key Laboratory of Bioreactor Engineering, East China University of Science and Technology, Shanghai, China; ^2^ Shanghai Key Laboratory of New Drug Design, School of Pharmacy, East China University of Science and Technology, Shanghai, China; ^3^ Center of Translational Biomedical Research, University of North Carolina at Greensboro, Greensboro, NC, United States; ^4^ College of Life Sciences, Xinyang Normal University, Xinyang, China

**Keywords:** survivin, P-Glycoprotein, CRISPR/Cas9-based Tet-off system, PI3K/Akt/mTOR pathway, MCF-7 cells, multi-drug resistance

## Abstract

Quantitative analysis and regulating gene expression in cancer cells is an innovative method to study key genes in tumors, which conduces to analyze the biological function of the specific gene. In this study, we found the expression levels of Survivin protein (*BIRC5*) and P-glycoprotein (*MDR1*) in MCF-7/doxorubicin (DOX) cells (drug-resistant cells) were significantly higher than MCF-7 cells (wild-type cells). In order to explore the specific functions of *BIRC5* gene in multi-drug resistance (MDR), a CRISPR/Cas9-mediated knocking-in tetracycline (Tet)-off regulatory system cell line was established, which enabled us to regulate the expression levels of Survivin quantitatively (clone 8 named MCF-7/Survivin was selected for further studies). Subsequently, the determination results of doxycycline-induced DOX efflux in MCF-7/Survivin cells implied that Survivin expression level was opposite to DOX accumulation in the cells. For example, when Survivin expression was down-regulated, DOX accumulation inside the MCF-7/Survivin cells was up-regulated, inducing strong apoptosis of cells (reversal index 118.07) by weakening the release of intracellular drug from MCF-7/Survivin cells. Also, down-regulation of Survivin resulted in reduced phosphorylation of PI3K, Akt, and mTOR in MCF-7/Survivin cells and significantly decreased P-gp expression. Previous studies had shown that PI3K/Akt/mTOR could regulate P-gp expression. Therefore, we speculated that Survivin might affect the expression of P-gp through PI3K/Akt/mTOR pathway. In summary, this quantitative method is not only valuable for studying the gene itself, but also can better analyze the biological phenomena related to it.

## Introduction

Quantitative analysis and controllable expression of genes is a novel method for studying key genes in tumor. This method not only helps to analyze the biological functions of specific genes themselves, but also better explains the biological phenomena related to them (Ede et al., 2016). For instance, Survivin (encoded by the *BIRC5* gene) is the ever-reported smallest inhibitor of apoptosis proteins (IAPs) (Garg et al., 2016; Khan et al., 2017). Its involvement in the occurrence and development of chemoresistance of tumors has been reported by some researchers (Shi et al., 2007; Liu et al., 2010; Souza et al., 2011; Warrier et al., 2020). Survivin expression was significantly higher in drug-resistant breast cancer cells than that of parent cells. The dominant-negative mutant of Survivin could increase the sensitivity of breast cancer cells to DOX, which suggested that Survivin was associated with tumor multi-drug resistance (MDR) (Xu et al., 2012). Therefore, in order to deeply explore the role of Survivin in MDR, it would be very helpful to develop a system that regulates the expression of *BIRC5* in real time. Meanwhile, the link between Survivin and chemotherapy resistance can be further explored.

**GRAPHICAL ABSTRACT:**
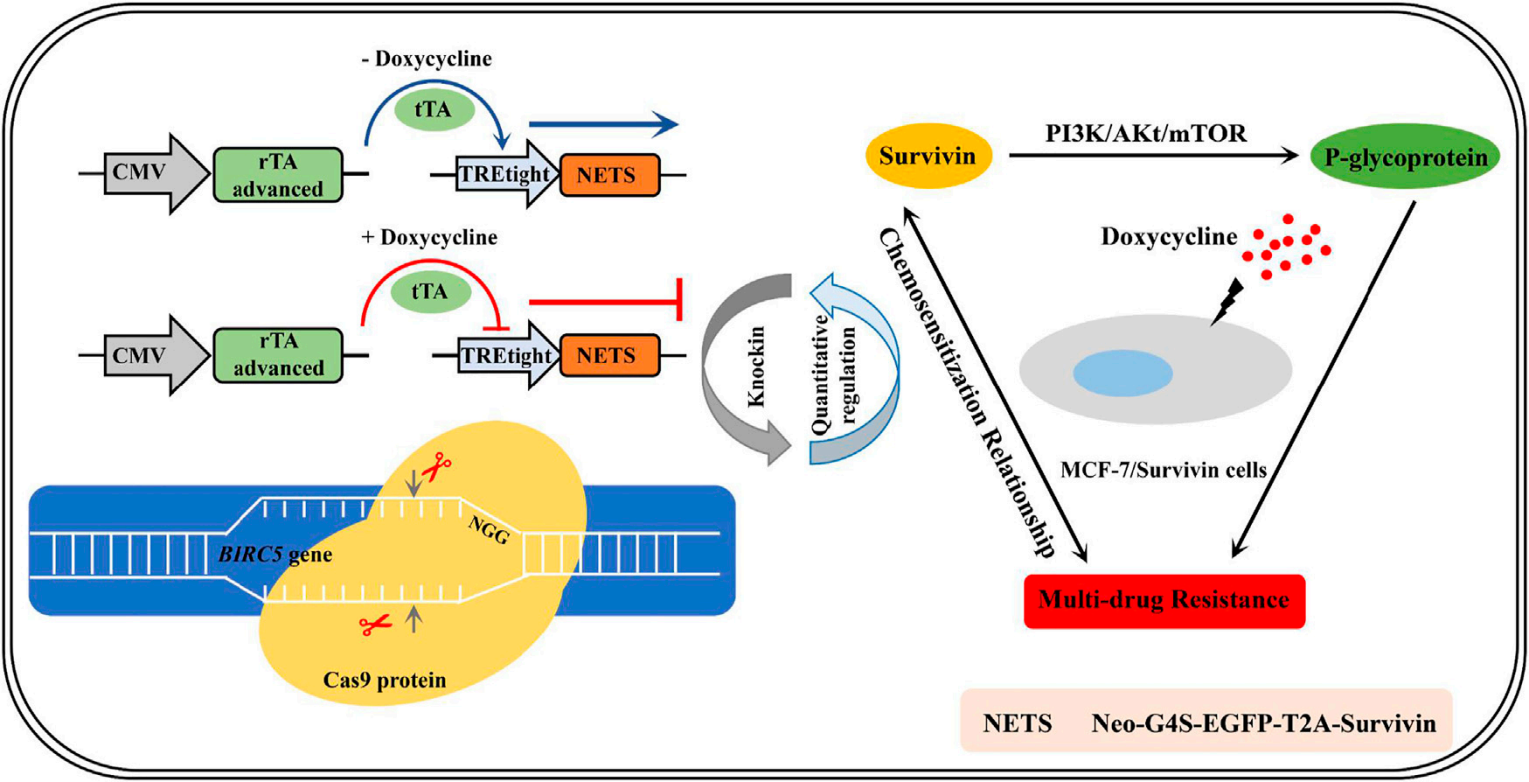
The establishment of quantitatively regulated Survivin-expressing cell lines enabled the exploration of chemosensitization mechanism in breast cancer cells.

Chemotherapy is a widely-adopted medication for many cancers that constitute a severe threat to our health (Rehman et al., 2021). Unfortunately, multi-drug resistance (MDR) of cancer cells, the phenomenon that tumor cells are insensitive to the treatment of one or more drugs, is a significant cause of cancer chemotherapy failure (Pan et al., 2016; Ramirez et al., 2016; Rathore et al., 2017; Adamska et al., 2018; Norouzi-Barough et al., 2018). Therefore, studying the MDR mechanism will tremendously contribute to guaranteeing existing medication efficiency (e.g., chemotherapy) and discovering novel cancer therapy. A generally recognized cause of cancer MDR is the expression of a class of energy-dependent efflux pump proteins called ATP-binding cassette (ABC) transporters (Eckford and Sharom, 2009; Zappe and Cichna-Markl, 2020). These proteins pump chemotherapeutic drugs that have already entered cancer cells to the extracellular environment by consuming ATP, leading to the emergence of MDR in tumor cells (Szakács et al., 2006; Zappe and Cichna-Markl, 2020). Some well-studied MDR proteins include P-glycoprotein (P-gp) (Tsubaki et al., 2012; Murakami et al., 2020), multi-drug-associated protein 1 (MRP1) (Johnson and Chen, 2017), breast cancer resistance protein (BCRP) (Wang et al., 2013; Safar et al., 2019), and lung resistance protein (LRP) (Kulsoom et al., 2019). Among these proteins, the high expression of P-gp is believed to be the primary cause for the MDR of cancer cells (Adamska et al., 2018).

The overexpression also supported the possible involvement in MDR of Survivin and P-gp in MDR cancerous cells (Liu et al., 2010; Tsubaki et al., 2012; Wang et al., 2015; Wang et al., 2017). Reis *et al.* (Reis et al., 2011) found that Survivin and P-gp were associated and highly expressed in the late phase of chronic myeloid leukemia. Liu and his colleagues showed that Survivin transcription was related to P-gp overexpression in the MDR MCF-7 cells (Liu et al., 2008). Tsubaki *et al.* (Tsubaki et al., 2012) reported that Survivin and P-gp could interfere with the activity of Caspase-8/3 and protect cells from apoptosis caused by chemotherapy. Survivin and P-gp are in the cytoplasm and cell membrane, respectively (Garg et al., 2016; Murakami et al., 2020). Shi *et al.* (Shi et al., 2007) found that Survivin did not directly interact with P-gp. However, it is still unclear how the expression of Survivin affects the expression of P-gp and thus the MDR of tumor cells to chemotherapy drugs.

Many anticancer drugs could down-regulate the expression of P-gp through the PI3K/Akt/mTOR pathway (Chen et al., 2015; Wang L. et al., 2016; Deng et al., 2017), thereby enhancing the anticancer efficacy of chemotherapy drugs (Zeng et al., 2018; Yu et al., 2020). Alike these drugs, Survivin also regulated the PI3K/Akt/mTOR pathway (Sandra and Khosravi-Far, 2014; Sandra, 2018). Guo *et al.*(Guo et al., 2019) proved that Ubenimex reversed the MDR of gastric cancer cells, accompanied by downregulation of Survivin expression. Ubenimex also downregulated membrane transport proteins’ expression (including P-gp) by inhibiting phosphorylation in the PI3K/AKT/mTOR pathway (Guo et al., 2019). These facts prompted us to study the connection amongst Survivin, P-gp, and PI3K/Akt/mTOR pathway in the MDR of tumor cells.

In the present study, CRISPR/Cas9-mediated knocking-in tetracycline (Tet)-off technology was used to precisely edit the *BIRC5* gene to quantitatively control the expression of Survivin in breast cancer cells MCF-7. As expected, the chemoresistance of the breast cancer cell was successfully reversed. Our findings imply that reversal of MDR may be realized by down-regulating P-glycoprotein level through control on Survivin expression, which provides a theoretical basis for the development of more effective or new cancer treatment options. Also, this quantitative method provides new insights for studying the biological functions of key genes in tumors.

## Materials and Methods

### Materials

PSpCas9(BB)-2A-GFP (pX458, Addgene, #48138) and pSpCas9(BB)-2A-Puro (pX459, Addgene, #48139) were purchased from Addgene (http://www.addgene.org). 3-(4, 5-dimethylthiazol-2-yl)-2, 5-diphenyltetrazolium bromide (MTT) was supplied by Aladdin Industrial Corporation (Shanghai, China). P-gp rabbit polyclonal antibody, PI3K mouse monoclonal antibody, mTOR mouse monoclonal antibody, Akt rabbit polyclonal antibody, p-PI3K rabbit polyclonal antibody, p-mTOR rabbit monoclonal antibody, and p-Akt mouse monoclonal antibody were products of Proteintech Group (Chicago, United States). 5-FU, DOX, paclitaxel, verapamil, and doxycycline were obtained from Sigma-Aldrich (Shanghai, China). All other reagents used in this study were of analytical grade and purchased from Sigma-Aldrich. Human breast cancer cell lines MCF-7 and MCF-7/DOX were purchased from the Type Culture Collection Committee of the Chinese Academy of Sciences (Shanghai, China). Fetal Bovine Serum (FBS), Roswell Park Memorial Institute 1,640 medium (RPMI 1640), Dulbecco’s Modified Eagle Medium (DMEM), and penicillin-streptomycin (10,000 U/mL penicillin and 10 mg/ml streptomycin) were obtained from Thermo Fisher Scientific (Waltham, United States).

### Targets Design and sgRNA Plasmids Construction for CRISPR/Cas9-Based Genome Editing

The sgRNA targeting *BIRC5* gene was selected with the help of the online program SYNTHGO (https://design.synthego.com) by taking the score and off-target possibility of all candidate sites. The designed sgRNA was then generated by annealed oligos and cloned into the *Bbs* I site of a constitutive mammalian P_U6_-driven sgRNA expression vector (pX458/pX459) (see Supplementary Figure S1 and Supplementary Table S1 for more details).

### Construction of Linear Expression Cassette

The linear expression cassette consisted of the following main components: a Tet-off system enabling the quantitatively regulated Survivin expression under a tight tetracycline response element (TRE-tight) and an advanced tetracycline-regulated transcriptional activator (tTA-advanced). The Survivin expression system mainly consisted of the target gene encoding Survivin and the reporter genes, including the neomycin resistance gene (Neo) and enhanced green fluorescent protein (EGFP) gene. The Neo and EGFP genes were linked by the flexible peptide (G4S) sequence favoring the correct folding of these proteins after expression. Besides, the self-splicing peptide T2A was introduced between the reporter gene (Neo-G4S-EGFP) and the target gene (Survivin), facilitating the generation of Survivin from Neo-G4S-EGFP. The final structure of the construct wasNeo-G4S-EGFP-T2A-Survivin (NETS). Pair primers used for obtaining the micro homologous linear expression cassette CMV-Tet-off-Neo-G4S-EGFP-T2A-Survivin-BGH Poly(A) (Tet-off-NETS) were listed in Supplementary Table S2.

### Cell Culture and Transfection

MCF-7 and MCF-7/DOX cells were cultivated in DMEM and RPMI 1640 supplemented with 10% (v/v) FBS, 1% penicillin-streptomycin, and 1 μg/ml DOX in incubator with CO_2_ (5% atmosphere) at 37 °C. After confluent growth of 80–90%, cells were trypsinized and seeded into plates. According to the manufacturer’s protocol, cells were transfected with Lipofectamine3000 (Thermo Fisher Scientific, United States). Briefly, MCF-7 cells at a density of 2 × 10^5^ were plated to a 24-well plate and incubated in 5% CO_2_ atmosphere at 37 °C for 24 h. Subsequently, 500 ng constructed plasmids with 1 μl P3000 and 0.75 μl Lipofectamine3000 mixtures were added into each well for co-transfection. The transfection experiments were conducted in triplicates. Subsequent experiments were performed at 48 h after transfection.

### T7E1 Assay

According to the manufacturer’s instructions, genetic DNA was extracted from MCF-7 cells using the TIANamp Genomic DNA Extraction Kit (TIANGEN, Beijing, China). The genomic region containing the target sites was amplified using the 2× Taq Plus Master Mix II (Dye Plus) DNA polymerase (Takara, Japan). The PCR amplicons were purified with HiPure Gel Pure Micro Kits (Magen, Guangzhou, China). Purified PCR products (500 ng) were mixed with 2 μl of 10 × PCR buffer for restriction enzyme and ultrapure water to a final volume of 20 μl and reannealed (94°C, 2 min; 98°C, 10 s, 65–55 (−0.1°C per cycle), 30 s; 72°C, 30 s, 10 cycles; 98°C, 10 s, 55°C, 30 s, 72°C, 30 s, 20 cycles; 72°C, 7 min) to form heteroduplex DNA. After reannealing, the heteroduplex DNA was treated with 0.25 μl of T7E1 (New England Biolabs, United States) for 1 h at 37°C and then analyzed by 2% agarose gel electrophoresis. Gels were stained with GelRed and imaged with Tanon 3,500 gel imaging system (Shanghai, China). Relative band intensities were calculated by ImageJ software. Indel percentage was determined by Equation 1, in which the “*a*” indicates the integrated intensity of the undigested PCR product, and *b* and *c* are the integrated intensities of each cleavage product.

$$\left(1\right)\text{ Indel }\left(\text{\%}\right)=\left(1-\left(1-\left(\text{b}+\frac{\text{c}}{\text{a}}+\text{b}+\text{c}\right)\right)/2\right)\times 100\text{\%}$$

### Monoclonal Screening

G418 (Sangon Biotech, Shanghai, China) was used to screen and enrich hybrid cell clones, and then the positive monoclonal cell lines were obtained by the limited dilution method. Briefly, the selected pX459-sgRNA (500 ng) and constructed linear expression cassette Tet-off-NETS (500 ng) were mixed with 1.5 μl Lipofectamine3000 and co-transfected into MCF-7 cells. After 48 h of transfection, the co-transfection efficiency of one group was detected by flow cytometer (FCM), while for another group, a fresh medium containing 2 mg/ml G418 was added to continue the cultivation. In the following 14 days, the medium was changed every 2 days. After a 14-day screening and enrichment with G418, the cells were digested to make a single-cell suspension and diluted to 0.5 cell/100 μl using a hemocytometer. Then, 200 μl of cell suspension was dispensed to each well of a 96-well plate and cultured in an incubator. After 48 h, a fresh medium containing 1 mg/ml G418 was used to replace the old one. The cultivation continued for another week with a medium change every 3 days. About 1 week later, the monoclonal wells were screened with an inverted microscope (Olympus Corporation, Japan). The positive clones were further amplified, passaged, and stored. The positive clone with the highest Survivin expression level was named MCF-7/Survivin. 5’/3’ junction PCR was performed to detect Non-Homologous End Joining (NHEJ)-mediated integration event using KOD-FX (TOYOBO, Japan). Primers used for the detection of NHEJ-mediated integration were listed in Supplementary Table S2.

### Verification of the Performance of the Tet-off Regulation System

MCF-7/Survivin cells were seeded in a 6-well plate at a density of 2 × 10^5^ cells/well, and cultured in an incubator at 37°C, for 24 h (5% CO_2_ atmosphere). After the incubation, 2 ml of DMEM complete medium containing different concentrations of doxycycline (0, 2.25, 22.5, 112.5, 225, 1,125, 2,250, 4,500 and 6,750 nmol/L) was added to each well. After 48 h incubation, the average fluorescence intensity of EGFP in the cells was detected by FCM.

### MTT Assay

MTT assay was used to determine the cytotoxicity of DOX (Cell viability and IC_50_). MCF-7/Survivin cells, and wild-type MCF-7 cells (WT MCF-7 cells) (2 × 10^5^ cells/well) were seeded into a 96-well plate, and cultured overnight before incubating with doxycycline (0, 2.25, 22.5, 225, 1,125, 2,250, and 4,500 nmol/L) and DOX (0, 0.1, 1, 5, 10, and 50 μmol/L) for 24 h (add separately or in pairs). After that, 20 μl of MTT solution (5 mg/ml) was added to each well and incubated for another 4 h at 37°C. The produced formazan was dissolved in dimethyl sulfoxide (DMSO, 150 μl), and the absorbance was detected at 490 nm by a microplate reader (Biotek Instruments Inc., United States). Analyze the data to determine its cell viability and IC_50_ value. Meanwhile, the drug resistance index (RDI) and reversal index (RI) were calculated based on the IC_50_ values of MCF-7/Survivin and WT MCF-7 cells.

$$RDI=MCF-7/Survivin\;\;IC_{50}\;/\;WT\;MCF-7\;\;IC_{50}$$

$$RI=IC_{50}\;\left(without\;doxycycline\;treatment\right)\;/\;IC_{50}\;\left(with\;doxycycline\;treatment\;\right)$$

### Apoptosis Assay

The cell apoptosis was quantified by Annexin V-FITC/PI assay. MCF-7/Survivin cells (2 × 10^5^ cells/well) were cultured overnight in 6-well plates and incubated with doxycycline (0, 22.5, 225, and 2,250 nmol/L) and/or DOX (0, 1, and 5 μmol/L) for 24 h. The harvested cells were washed with PBS, resuspended in 200 µl of binding buffer, and stained at room temperature in the dark with Annexin V-FITC and propidium iodide (PI) (Sangon Biotech, China). The cell apoptosis was analyzed by FCM with FITC channel (λ_ex_ = 488 nm and λ_em_ = 525 nm) and PI channel (λ_ex_ = 535 nm and λ_em_ = 615 nm).

### Drug Efflux Analysis of DOX

MCF-7/Survivin cells (2 × 10^5^ cells/well) were cultured overnight in 6-well plates and incubated with doxycycline (0, 22.5, 225, 1,125, and 2,250 nmol/L) and DOX (0, 1, and 5 μmol/L) for 24 h. Then the fresh DMEM medium containing 2 μmol/L DOX was added to each well, and the plate was incubated for another 4 h in the dark. Afterward, the cells were harvested and washed with PBS. After being resuspended in 200 µl PBS, cells were analyzed by FCM.

### RNA-Sequence (RNA-Seq) Assay

Transcriptome data are all from the oncomine database, and its website is https://software.oncomine.com/resource/login.html. We chose Garnett cell line and grouped them by DOX sensitivity (cell line). A total of 732 cell lines, including No value (415), DOX resistant (92), DOX intermediate sensitivity (126), and DOX sensitive (99), were used for data analysis. Meanwhile, the expression levels of *BIRC5* and *MDR1* were compared in the database. And *BIRC5* coexpressed with *MDR1* in Garnett cell line grouped by DOX sensitivity (cell line), log2 median-centered intensity.

### Quantitative Real-Time PCR (qRT-PCR) Assay

Total RNA of cells was extracted using the RNAiso Plus kit (Takara, Japan). A total of 500 ng of RNA was reverse transcribed into cDNA using a PrimeScript RT Reagent Kit with the genomic DNA Eraser (Takara, Japan). qRT-PCR reactions were performed on the LightCycler 96 real-time PCR instrument (BioRad, United States) using the SYBR Premix ExTaq. Program for qRT-PCR amplification was as follows: 95°C for 5 s, followed by 40 cycles at 95°C for 10 s, 55°C for 10 s, and 72°C for 20 s. The primers used were listed in Supplementary Table S2. Samples were normalized to the housekeeping gene β-actin as the endogenous control.

### Western Blotting

Western blotting was used to assess the expression of phosphorylated and unphosphorylated PI3K, Akt, and mTOR, as well as P-gp and Survivin proteins in MCF-7/Survivin cells. The cell lines (2 × 10^5^ cells/well) were cultured in 6-well plates and treated with different doxycycline concentrations (0, 2.25, 22.5, 225, and 2,250 nmol/L) for 24 h. Then, cells were lysed with lysis buffer, and the lysate was collected. After SDS-PAGE (12%), the samples were electro-transferred onto a pretreated PVDF membrane. The membrane was blocked using 3% non-fat milk (or 5% bovine serum albumin (BSA)) in Tris-buffer saline with Tween 20 (TBST) for 2 h at room temperature and incubated with antibodies at 4 °C overnight. Further incubation with the secondary antibody was performed for 1 h at room temperature after washing with TBST 3 times. Protein bands were visualized with the ultrasensitive ECL chemiluminescence kit (Sangon Biotech, Shanghai, China). Protein quantification was performed with ImageJ (1.51K).

### Statistical Analysis

All data were presented as the mean ± standard deviation (SD). The significance of differences between groups was evaluated by one-way ANOVA with LSD (Least significant difference) post hoc test. *p* < 0.05 was defined as statistically significant difference.

## Result

### Expression of Survivin and P-Gp Was Positively Correlated with the Chemotherapy Drug Resistance of MCF-7/DOX Cells

**FIGURE 1:**
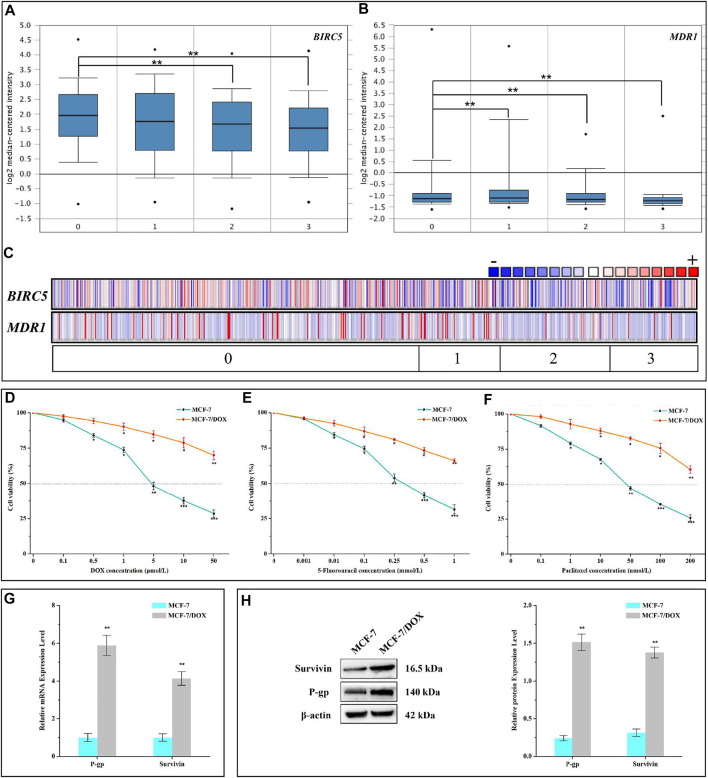
Resistance of MCF-7/DOX cells to chemotherapy drugs. **(A–C)** *BRIC5* and *MDR1* expression in Garnett cell line grouped by DOX sensitivity (cell line). 0, 1, 2, and 3 represent No value (n = 415), DOX Resistant (n = 92), DOX Intermediate Sensitivity (n = 126), and DOX Sensitive (n = 99). The points in A and B represent the extreme outlier of the corresponding data. **(D–F)** Viabilities of MCF-7 and MCF-7/DOX cells treated with different concentrations of DOX, 5-FU, and paclitaxel. **(G)** mRNA and **(H)** protein levels of Survivin and P-gp in MCF-7 and MCF-7/DOX cells. D-H were performed 3 parallel biological replicates. Data of D-H were expressed as mean ± SD (n = 3). *p < 0.05, **p < 0.01, and ***p < 0.001.

The leading cause of chemotherapy failure in cancer patients is believed to be the development of MDR (Szakács et al., 2006; Adamska et al., 2018; Norouzi-Barough et al., 2018). Based on the online transcriptome database, we found that compared with the control group, the expression of *BIRC5* in resistant lines was basically unchanged, while the expression level of sensitive lines was significantly down-regulated (Figure 1A). Meanwhile, *MDR1* was highly expressed in resistant lines and low in sensitive lines (Figure 1B). Subsequently, the co-expression analysis of the two was also carried out (Figure 1C). These results potentially indicated that *BIRC5* and *MDR1* were related to MDR. According to our experiment, the half-maximal inhibitory concentration (IC_50_) of DOX to MCF-7/DOX and MCF-7 cells was 310.10 ± 17.10 μmol/L and 6.15 ± 0.64 μmol/L, respectively. In the presence of DOX, the cell viability of MCF-7/DOX cells was significantly higher than that of wild type (WT) MCF-7 cells (Figure 1D). The resistance index of MCF-7/DOX cells was 50.46 (Table 1). These results indicated that MCF-7/DOX cells had more robust resistance to DOX. Meanwhile, MCF-7/DOX cells also had strong cross-resistance to 5-FU (Figure 1E) and paclitaxel (Figure 1F) with a resistance index of 22.97 and 34.29, respectively. The mRNA and protein levels of Survivin and P-gp in MCF-7 and MCF-7/DOX cells were further analyzed by qRT-PCR and western blotting. As shown in Figures 1G,H, the mRNA and protein levels of Survivin and P-gp in MCF-7/DOX were up-regulated significantly (*p* < 0.01). Compared with the MCF-7 cells, the mRNA levels of Survivin and P-gp were 4.14- and 5.90-fold and the protein levels were 4.38- and 6.26-fold. These results indicated that the resistance of MCF-7/DOX cells to chemotherapy drugs was positively correlated with the high expression levels of Survivin and P-gp.

**TABLE 1:** IC_50_ value and RDI of DOX, 5-FU, and paclitaxel against MCF-7 and MCF-7/DOX cells.

Agents	IC_50_		
	MCF-7	MCF-7/DOX	RDI
Doxorubicin (μmol/L)	6.15 ± 0.64	310.10 ± 17.10	50.46
5-Fluorouracil (mmol/L)	0.32 ± 0.04	7.40 ± 0.91	22.97
Paclitaxel (nmol/L)	29.47 ± 2.84	1,010.26 ± 113.49	34.29

Data were expressed as mean ± SD (n = 3).

### Design, Construction, and Screening of sgRNA Targeting *BIRC5* Gene for Efficient Genome Editing

**FIGURE 2:**
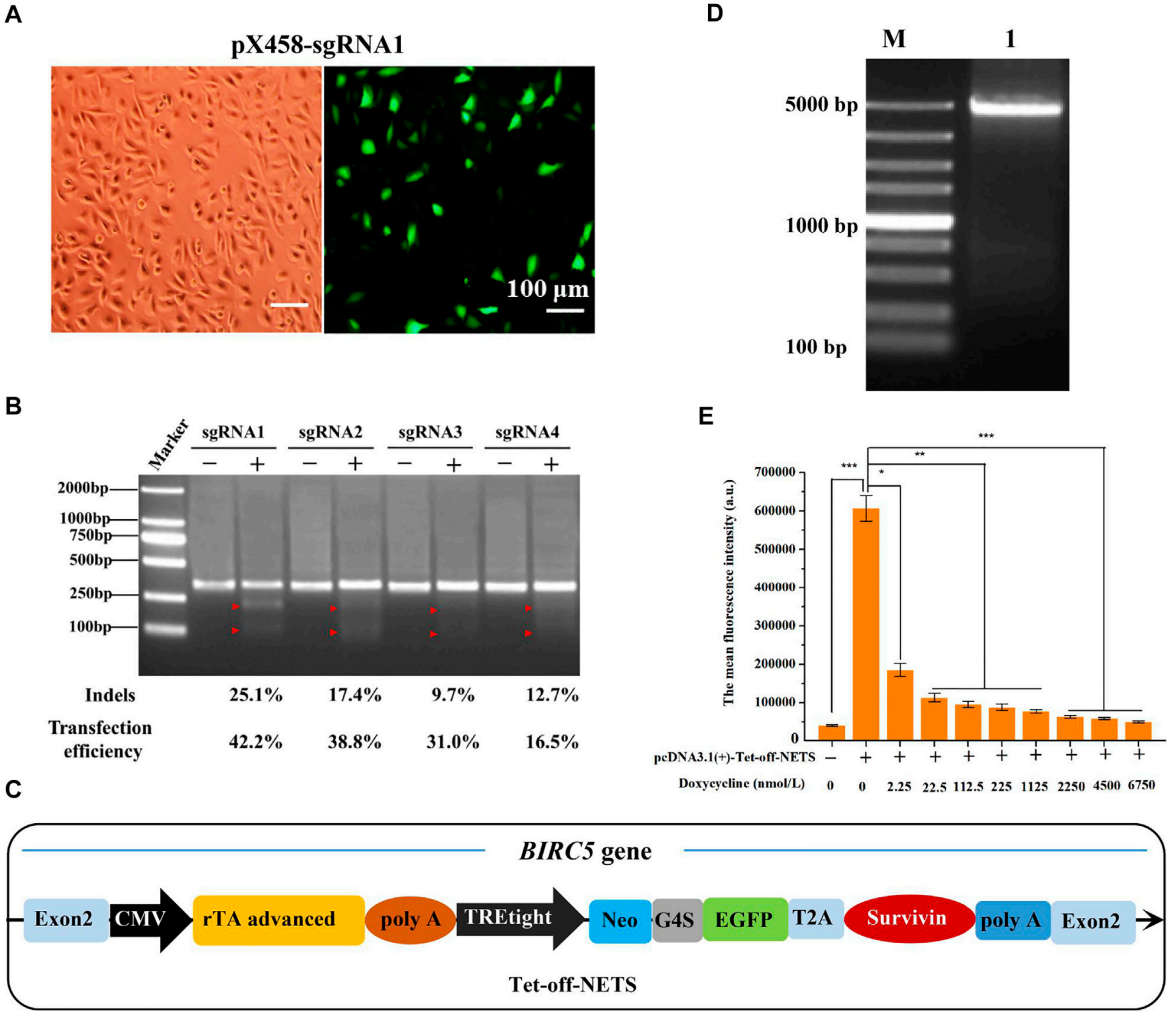
Selection of sgRNA targeting *BIRC5* gene for genome editing and construction of linear expression cassette. **(A)** Fluorescence images of MCF-7 cells transfected with pX458-sgRNA1 plasmid. All images were taken at ×100 magnification (Bar = 100 μm). **(B)** Agarose gel image of T7E1 assay to detect gene editing efficiency of different sgRNA. **(C)** Schematic diagram of the linear expression cassette (Tet-off-NETS). The heterologous Tet-off-NETS contains the Tet-off regulatory system and expression system (NETS). **(D)** Agarose gel electrophoresis of PCR amplification of linear expression cassette Tet-off-NETS. Lane M, DL5000 Marker; lane 1, Tet-off-NETS. **(E)** The mean fluorescence intensity of MCF-7 cells transfected with pcDNA3.1 (+)-Tet-off-NETS and treated with different concentrations of doxycycline for 24 h. E was performed 3 parallel biological replicates. Data were expressed as mean ± SD (n = 3). *p < 0.05, **p < 0.01, and ***p < 0.001.

We aimed to construct a stable cell line in which the background expression of naturally existing *BIRC5* gene (including different forms of spliceosomes) was eliminated. To do so, we knocked out all the background *BIRC5* spliceosomes in MCF-7 cells and then knocked in the exogenous *BIRC5* gene into MCF-7 cells through CRISPR/Cas9 genome editing technology. By analyzing all spliceosomes of *BIRC5*, we found that exon 2 universally existed. We used the online tool to aid the sgRNA design and selection. Among all the 25 recommended sgRNAs targeting exon 2, four sgRNAs with the highest scores (an indicator of high efficiency and low off-target effects) were selected (Supplementary Figure S1A). Subsequently, the synthesized sgRNA oligos were annealed into double-stranded DNA and cloned into vectors pX458 (Supplementary Figure S1B). The resultant plasmids pX458-sgRNA1, pX458-sgRNA2, pX458-sgRNA3, and pX458-sgRNA4 were used in sgRNA screening and co-transfection experiments. The enhanced green fluorescent protein (EGFP) gene was also included in the recombinant plasmid pX458-sgRNA to facilitate the analysis of transfection efficiency in MCF-7 cells. The transfection efficiency of empty vector pX458 was 44.8%. By contrast, the transfection efficiency of the four sgRNA-containing plasmids was slightly lowered, reaching 42.2, 38.8, 31.0, and 16.5%, respectively (Figure 2A, Supplementary Figure S2A,S2B). Also, the T7E1 restriction enzyme digestion experiment was carried out to verify whether the sgRNA and Cas9 enzymes could target the *BIRC5* gene and perform efficient site-directed cutting (Supplementary Figure S3). As shown in Figure 2B, the target DNA was digested into two fragments by the T7E1 enzyme, indicating that all the four designed pX458-sgRNA vectors could be used in the genome editing targeting the *BIRC5* gene. The indels introduced by the four recombinant plasmids were 25.1, 17.4, 9.7, and 12.7%, respectively. These results demonstrated that the plasmid pX458-sgRNA1 had the highest cutting efficiency and transfection efficiency (42.2%). After these screening processes, the sgRNA1 was constructed into plasmid pX459, and the resultant plasmid pX459-sgRNA1 was used for subsequent experiments.

### Construction of the Tet-Off-NETS Expression Cassette

The desired expression cassettes mainly included the Tet-off regulatory system and the NETS expression system (Figure 2C). The needed gene fragments were prepared by PCR amplification and then fused by overlap extension PCR to create the NETS expression system (Supplementary Figures S4A,S4B). The obtained expression cassette was designated as Tet-off-NETS (4,085 bp, Figure 2D and Supplementary Figure S4C). A sequence homologous to the *BIRC5* gene cleavage site (9 bp) was included for efficient recombination (Nakade et al., 2014).

### Verification of the Single-Plasmid Tet-Off Regulatory System

To verify whether the Tet-off regulatory system could work properly, we tested the gene expression with doxycycline induction. MTT results showed that after MCF-7 cells were treated with different concentrations of doxycycline (0, 2.25, 22.5, 112.5, 225, 1,125, 2,250, 4,500, 6,750 nmol/L) for 24, 48, and 72 h, the cell viability was higher than 90%. When the concentration reached 22,500 nmol/L, the cell viability was reduced to 68.90, 39.83, and 27.12% at 24, 48, and 72 h, respectively (Supplementary Figure S5A). Besides, the morphology of MCF-7 cells treated with 22,500 nmol/L doxycycline changed markedly (Supplementary Figure S5B). As aresult, when the concentration of doxycycline was greater than 6,750 nmol/ml, the cell viability of the three time periods decreased significantly. When the concentration of doxycycline was less than 6,750 nmol/ml, there was no significant difference in the cell viability of the three time periods, indicating that as long as the concentration of doxycycline was controlled within 6,750 nmol/ml and the treatment time was 24–72 h, it will basically not affect MCF-7 Cell viability. Therefore, in order to speed up the process of the experiment and make the results faster, we chosen 24 h as the incubation time of doxycycline. And the doxycycline concentration was set in the range of 0–6,750 nmol/L to achieve a quantitative control on the downstream genes’ expression.

The expression of EGFP by doxycycline induction was analyzed by flow cytometry (FCM) with the single-plasmid Tet-off regulatory system. In the absence of doxycycline, the plasmid harboring the Tet-off regulatory system could transfect MCF-7 cells with an efficiency of 33.9%. When the doxycycline concentration was in the range of 0–6,750 nmol/L, the transfection efficiency decreased in a concentration-dependent manner (Supplementary Figure S5C). For instance, when doxycycline concentration was 2,250 nmol/L, the transfection efficiency was only 0.72%. The average fluorescence intensity of MCF-7 cells also exhibited a similar tread (Figure 2E). Based on these results above, we concluded that the Tet-off regulatory system in the expression cassette could quantitatively control downstream target genes’ expression through doxycycline induction.

### Screening and Identification of Positive Monoclonal Cell Line MCF-7

PX459-sgRNA1 and the linear expression cassette Tet-off-NETS were co-transfected into MCF-7 cells to construct a positive monoclonal cell line. The co-transfection efficiency was found to be 25.7% (Supplementary Figure S6A). The IC_50_ of G418 on MCF-7 cells was determined as 2.09 ± 0.18 mg/ml in MTT assay (Supplementary Figure S6B). Therefore, 2 mg/ml G418 was selected as the drug concentration to screen the positive monoclonal cell lines. After 48 h of co-transfection, 2 mg/ml G418 was used for 14-days to enrich positive cells (Supplementary Figure S6C). Subsequently, positive monoclonal cells were screened with the series dilution method in 96-well plates. The EGFP-positive monoclonal cells were selected and cultured. Integration of the expression cassette was identified by junction PCR (Supplementary Figure S6D). Among the predicted possible off-target sites of sgRNA1 (Supplementary Table S1), two highly homologous target sites were analyzed using junction PCR amplification with primers listed in Supplementary Table S2. No evident amplification was observed in junction PCR, which indicated that sgRNA1 did not cut at the predicted potential off-target sites (Supplementary Figure S6D).

**FIGURE 3:**
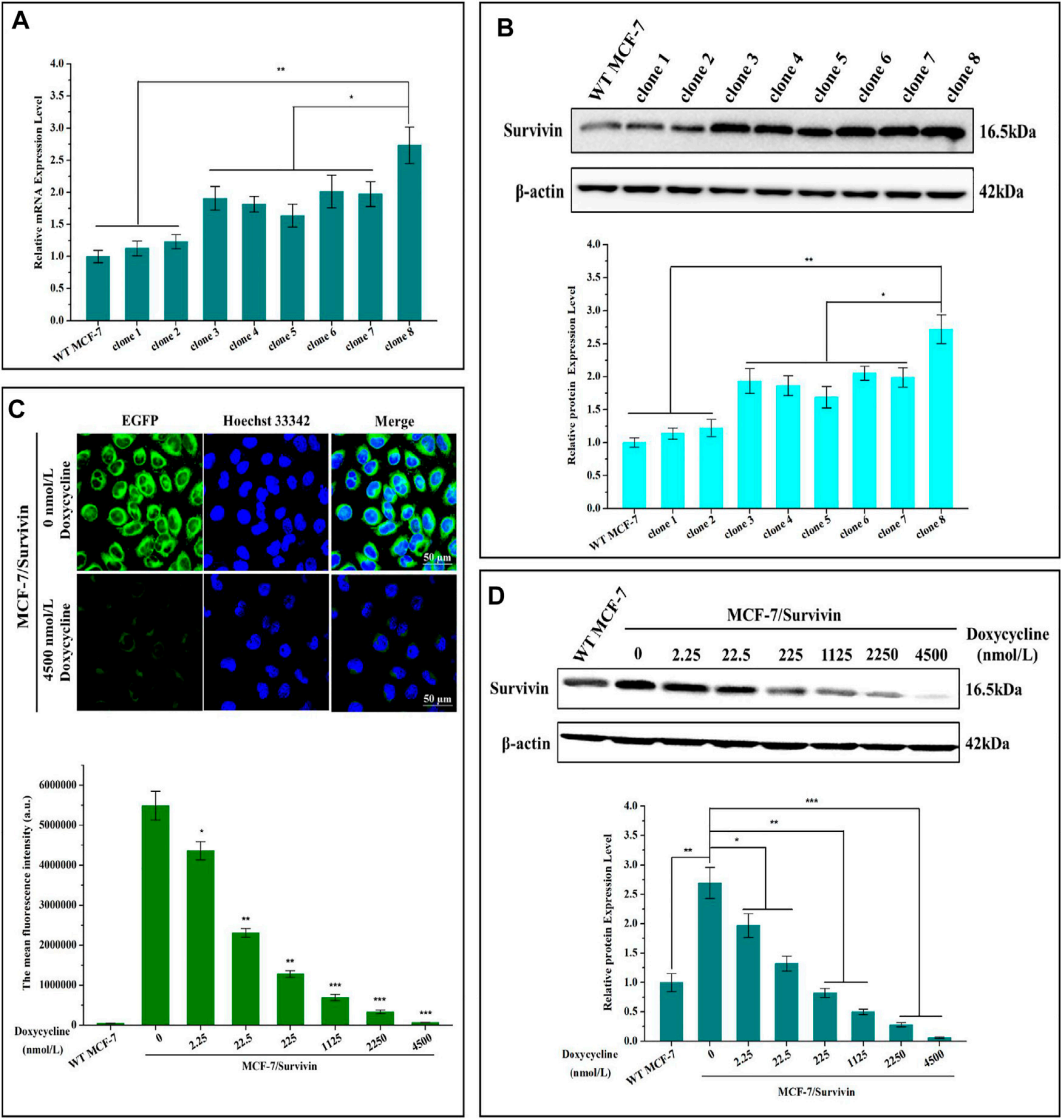
Screening and verification of positive monoclonal cell line MCF-7/Survivin. **(A)** mRNA and **(B)** protein levels of Survivin in different positive monoclonal cell lines. **(C)** The fluorescence distribution of EGFP observed by CLSM. Green and blue colors indicated EGFP and Hoechst 33,342, respectively. (Bar = 50 μm). Histogram mean fluorescence intensity of cells detected by FCM. **(D)** Changes of Survivin expression in MCF-7/Survivin cells treated with different concentrations of doxycycline. A-D were performed 3 parallel biological replicates. Data were expressed as mean ± SD (n = 3). *p < 0.05 and **p < 0.01.

Eight monoclonal cell lines were selected based on junction PCR amplification. mRNA and protein levels of Survivin in these cells were quantified by qRT-PCR and western blotting. Results indicated that mRNA and protein levels of Survivin in the eight cell lines were higher than that of the WT MCF-7 cells, implying that the expression cassette was correctly integrated into exon 2 of the *BIRC5* gene of MCF-7 cells and could be correctly transcribed and translated. Moreover, the mRNA and protein levels of Survivin in clone 8 was significantly higher than those of the other seven monoclonal cell lines, reaching 2.73- and 2.72-fold that of WT MCF-7 cells, respectively (Figures 3A–D). Therefore, this clone was named MCF-7/Survivin and chosen for further study.

### Survivin Expression in MCF-7/Survivin Cells Could Be Quantitatively Regulated by Doxycycline

To verify that doxycycline could quantitatively regulate the expression of Survivin in MCF-7/Survivin cells, we detected the expression of EGFP by Confocal Laser Scanning Microscope (CLSM) and FCM. Intensive green fluorescence dominantly located in the cytoplasm of the MCF-7/Survivin cells was observed when doxycycline was absent (Figure 3C). When 4,500 nmol/L doxycyclines were added, the green fluorescence in the cells was significantly reduced. Besides, in the presence of 4,500 nmol/L doxycyclines, the average fluorescence intensity in MCF-7/Survivin cells was comparable to that of the WT MCF-7 cells. Quantitative regulation on the expression of EGFP in MCF-7/Survivin cells could be achieved by controlling doxycycline concentration.

The self-cleavable peptide T2A was introduced between the reporter gene Neo-G4S-EGFP and the target protein Survivin to ensure the formation of functional Survivin and Neo-EGFP. As shown in Supplementary Figure S6E, the cleavage efficiency of the T2A peptide reached 98.41%, indicating that Survivin and Neo-G4S-EGFP were dominantly expressed as two separate proteins after the self-cleavage of the T2A peptide. The doxycycline-regulated Survivin expression in MCF-7/Survivin cells was detected by western blotting. As shown in Figure 3D, the expression of Survivin in MCF-7/Survivin cells decreased as a response to the increase of the doxycycline concentration. After treated with 225 nmol/L doxycyclines for 24 h, the expression of Survivin in MCF-7/Survivin cells was close to the background level. When the doxycycline concentration increased to 4,500 nmol/L, the expression of Survivin decreased significantly and approached zero (*p* < 0.001), but it ccould still partially detect the expression of Survivin protein. Based on these results, we concluded that doxycycline could quantitatively regulate the expression of Survivin in the constructed MCF-7/Survivin cells.

### Survivin Enhanced the Resistance of MCF-7/Survivin Cells to DOX

**FIGURE 4:**
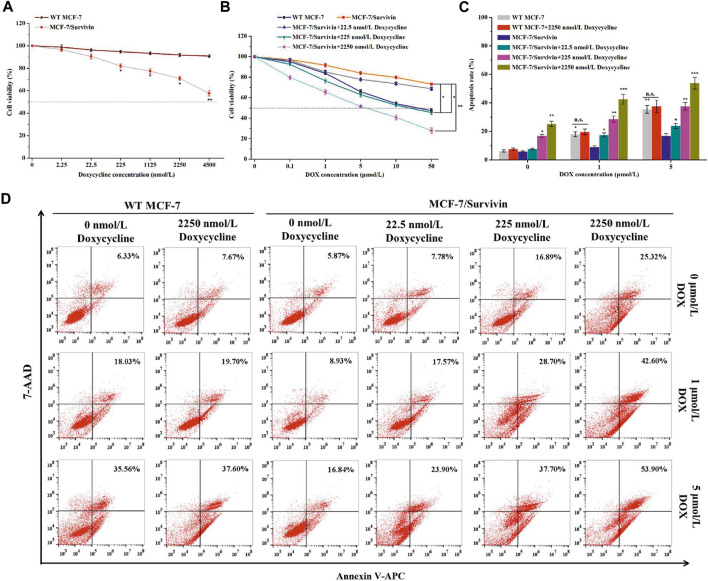
The resistance of MCF-7/Survivin cells to DOX. Cytotoxicity of **(A)** doxycycline and **(B)** DOX against MCF-7/Survivin cells detected by MTT assay. **(C)** Histogram of apoptosis rate of MCF-7/Survivin cells incubated with doxycycline and DOX for 24 h. **(D)** Apoptosis of MCF-7/Survivin and WT MCF-7 cells analyzed by FCM. A-C were performed 3 parallel biological replicates. Data were expressed as mean ± SD (n = 3). *p < 0.05, **p < 0.01, and ***p < 0.001. n. s. not significant.

Survivin expression in MCF-7/Survivin cells could be regulated by doxycycline. Subsequently, we analyzed the resistance of MCF-7/Survivin cells expressing different levels of Survivin to DOX. The cytotoxicity of doxycycline to MCF-7 (WT) and MCF-7/Survivin cells was tested by MTT assay. MCF-7 (WT) cells’ viability was greater than 90%, indicating that doxycycline did not affect cell proliferation. However, in MCF-7/Survivin cells, the cell viability gradually decreased with the increase of doxycycline concentration (Figure 4A). When the doxycycline concentration reached 4,500 nmol/L, the cell viability was reduced to 57.80 ± 2.40% (*p* < 0.01). As previously reported, doxycycline could change the tetR structure in the Tet-off regulatory system and influence the specific binding of tTA to tetO, thereby preventing the transcription of downstream target genes. Therefore, the low-level of Survivin in MCF-7/Survivin cells could inhibit cell proliferation and eventually led to cell apoptosis. The cell viability of MCF-7/Survivin cells treated with DOX was significantly higher than that of WT MCF-7 cells (*p* < 0.05), indicating that the high expression of Survivin in MCF-7/Survivin cells could enhance the resistance to DOX. The resistance index of MCF-7/Survivin cells to DOX was determined to be 21.96 (Table 2). When doxycycline was added, DOX inhibition on MCF-7/Survivin cell proliferation gradually increased (Figure 4B). When the doxycycline concentration reached 2,250 nmol/L, the cell viability was reduced to 27.96 ± 2.52% (*p* < 0.01). The drug resistance index was only 0.19 (Table 2). These data were in acceptable accord with our hypothesis: doxycycline could reverse the resistance of MCF-7/Survivin cells to DOX in 2,250 nmol/L doxycycline (reversal index 118.07).

**TABLE 2:** IC_50_ value, RDI, and RI of MCF-7/Survivin cells.

Cells	Doxycycline (nmol/L)	IC_50_ (μmol/L)	RDI	RI
WT MCF-7	0	24.63 ± 1.50	—	—
MCF-7/Survivin	0	540.64 ± 44.26	21.96	—
	22.5	315.30 ± 25.50	12.80	1.72
	225	20.46 ± 1.65	0.83	26.42
	2,250	4.58 ± 0.30	0.19	118.07

Data were expressed as mean ± SD (n = 3).

The effect of DOX on apoptosis of MCF-7/Survivin cells was further analyzed using the Annexin V-APC/7-AAD double-staining method (Figures 4C,D). There is no significant difference in apoptosis rates of MCF-7(WT) cells treated with 0–2,250 nmol/L doxycycline, indicating that doxycycline (up to 2,250 nmol/L) did not induce cell apoptosis. 24 h later, the apoptosis rates of MCF-7(WT) cells treated with 1 μmol/L and 5 μmol/L DOX, reached 18.03 ± 1.78% and 35.56 ± 2.72%, respectively. In the absence of doxycycline, after 24 h treatment with 1 μmol/L and 5 μmol/L DOX, the apoptosis rate of MCF-7/Survivin cells decreased by 9.10 and 18.72% compared with WT MCF-7 cells. The results demonstrated that MCF-7/Survivin cells could resist DOX-induced apoptosis. However, in the presence of 2,250 nmol/L doxycycline, the apoptosis rate of MCF-7/Survivin cells treated with 0, 1 and 5 μmol/L DOX for 24 h reached 25.35 ± 1.83% (*p* < 0.01), 42.60 ± 3.34% (*p* < 0.001), and 53.90 ± 4.11% (*p* < 0.001), respectively. A positive correlation between apoptosis rate and the inducer concentration was observed. The BIR domain of Survivin could interfere with apoptosis by inhibiting Caspase activity (Chiou et al., 2003). We deduced that the highly expressed Survivin in MCF-7/Survivin cells should be the cause for the resistance to DOX-induced apoptosis. When doxycycline was added, the expression of Survivin in MCF-7/Survivin cells was proportionally down-regulated, leading to a significant decrease in the DOX resistance of the cells and, consequently, more significant apoptosis.

### Down-Regulated Survivin Inhibited P-Gp Expression in MCF-7/Survivin Cells

**FIGURE 5:**
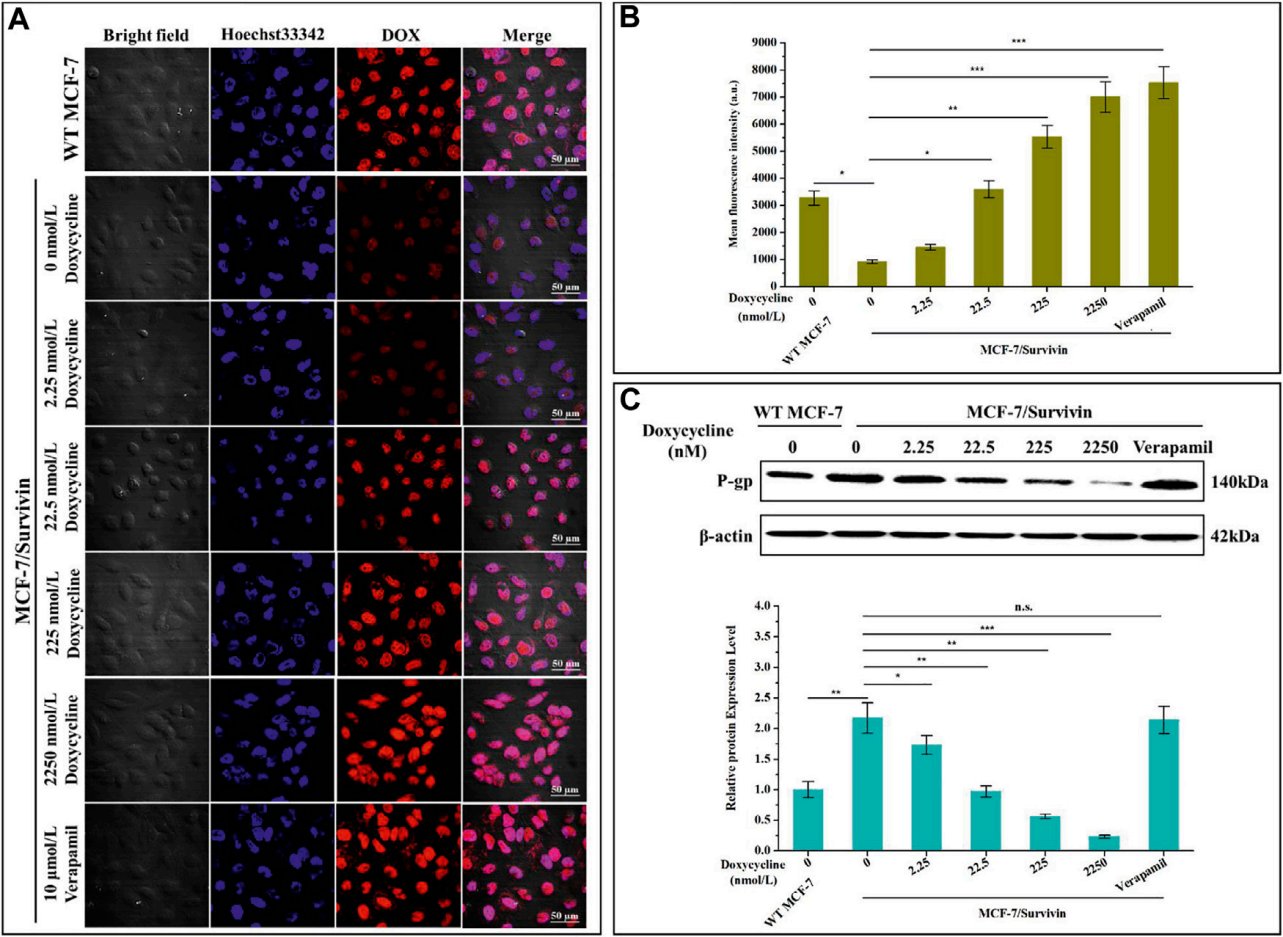
Determination of DOX efflux of MCF-7/Survivin cells induced by doxycycline. **(A)** Retention of DOX in MCF-7/Survivin cells observed by CLSM. Red and blue represent DOX and Hoechst 33,342, respectively. (Bar = 50 μm) **(B)** The mean fluorescence intensity of cells detected by FCM. **(C)** Western blotting detected P-gp expression in MCF-7/Survivin cells treated with doxycycline or verapamil. B-C were performed 3 parallel biological replicates. Data were expressed as mean ± SD (n = 3). *p < 0.05, **p < 0.01, and ***p < 0.001. n. s. not significant.

To better understand the potential mechanism by which Survivin enhanced the resistance of MCF-7/Survivin cells to DOX, we further studied the accumulation and retention of DOX in cells. In the absence of doxycycline, the red fluorescence of DOX in MCF-7/Survivin cells was significantly weaker than that in MCF-7 (WT) cells (Figure 5A). When doxycycline was added, the red fluorescence in MCF-7/Survivin cells gradually increased. After treatment with 2,250 nmol/L doxycycline, the intracellular red fluorescence was significantly higher than in the untreated MCF-7/Survivin cells. Meanwhile, the FCM results also suggested that, in the absence of doxycycline, the average fluorescence intensity of MCF-7 cells was 3.57-fold of that in MCF-7/Survivin cells (Figure 5B). However, the average fluorescence intensity of MCF-7/Survivin cells increased significantly as the concentration of doxycycline increased. Compared with untreated MCF-7/Survivin cells, treatment with 22.5, 225, and 2,250 nmol/L doxycycline could induce a 3.92, 6.03, 7.65 folds increase in intracellular fluorescence intensity, respectively. These results demonstrated that the down-regulation of Survivin in MCF-7/Survivin cells led to the accumulation and retention of DOX in MCF-7/Survivin cells.

To further confirm that the accumulation and retention of DOX in MCF-7/Survivin cells were correlated with DOX transport by P-gp in MCF-7/Survivin cells, the expression level of P-gp in these cells was quantified by western blotting. As shown in Figure 5C, when doxycycline was absent, the expression level of P-gp in MCF-7/Survivin cells was 2.17-fold that of MCF-7 (WT) cells. With the gradual increase of doxycycline concentration, the expression of P-gp in MCF-7/Survivin cells decreased significantly. Verapamil could only interfere with the transport function of P-gp and did not affect P-gp expression (Kimchi-Sarfaty et al., 2007; Peng et al., 2020). Therefore, verapamil (10 μmol/L) was used as a positive control. Compared with MCF-7/Survivin cells without doxycycline treatment, the intracellular fluorescence and average fluorescence intensity of MCF-7/Survivin cells treated with verapamil increased significantly (*p* < 0.001). As expected, the P-gp expression in MCF-7/Survivin cells treated with verapamil was comparable to that in untreated cells. Therefore, we believed that the P-gp expression level difference was not the primary explanation for the high concentration of intracellular DOX. In other words, the down-regulation of Survivin in MCF-7/Survivin inhibited the expression and thus the function of P-gp, thereby leading to the weakened export of DOX out of the MCF-7/Survivin cells.

### Down-Regulated Survivin Might Affect PI3K/Akt/mTOR Pathway to Decrease P-Gp Expression

**FIGURE 6:**
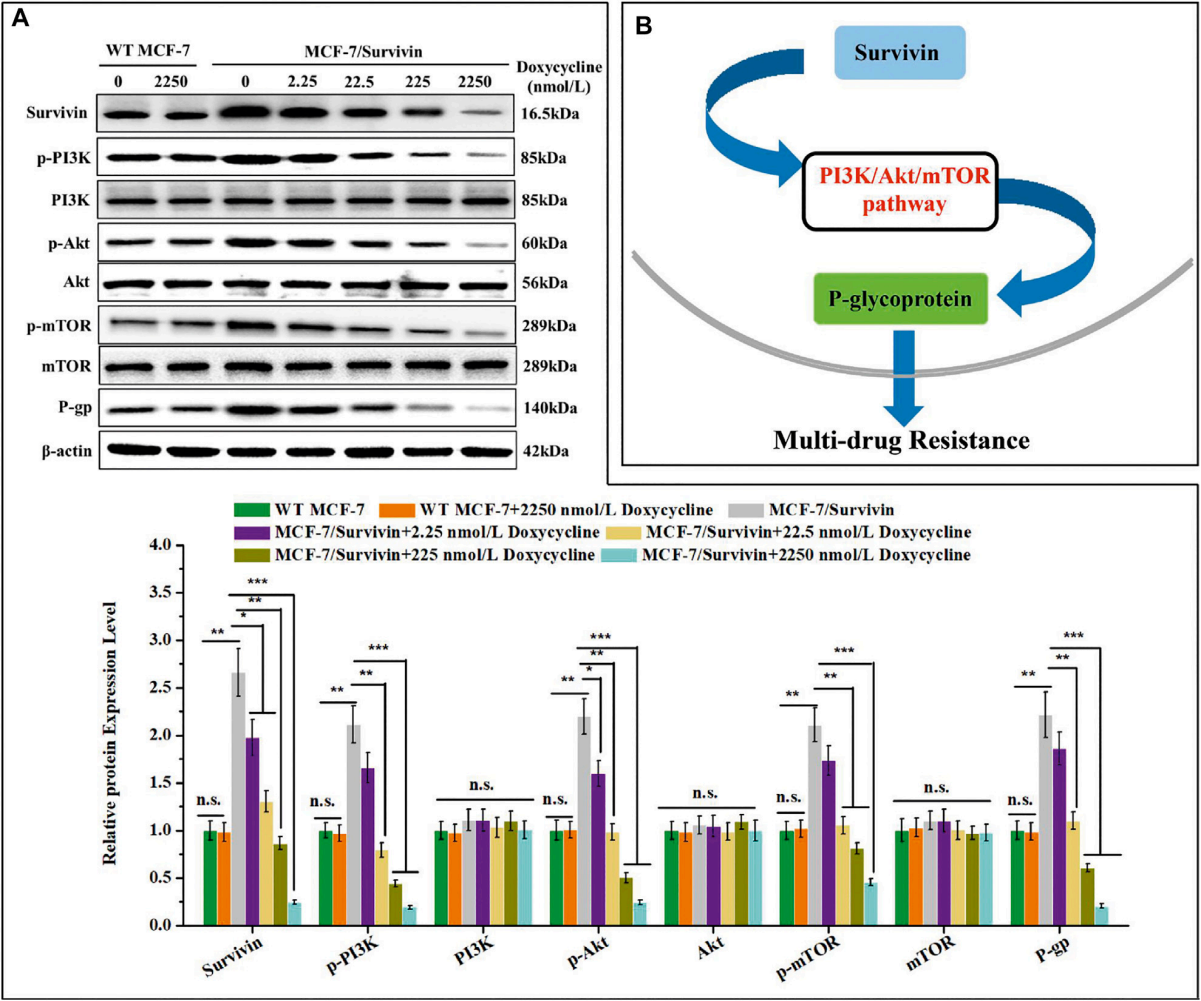
Synergistic effect of Survivin and P-glycoprotein expression might through PI3K/Akt/mTOR pathway. **(A)** The expression of Survivin, P-gp, and phosphorylated/unphosphorylated PI3K, Akt, and mTOR detected by western blotting. **(B)** Schematic diagram of the mechanism of Survivin affecting P-gp expression. A was performed 3 parallel biological replicates. Data were expressed as mean ± SD (n = 3). *p < 0.05, **p < 0.01, and ***p < 0.001. n. s. not significant.

Studies showed that Survivin could regulate the PI3K/Akt/mTOR pathway, thereby affecting P-gp expression (Guo et al., 2019). Therefore, the effect of Survivin level on the PI3K/Akt/mTOR pathway was studied with the MCF-7/Survivin cells in which the expression of Survivin was quantitatively regulated by doxycycline. In MCF-7(WT) cells, no significant difference in the expression of the related proteins in the doxycycline-treated cells and the control, indicating that the addition of doxycycline did not affect the protein expression in these cells **(** Figure 6A **)**. In MCF-7/Survivin cells, the expression level of Survivin without doxycycline treatment was significantly higher than that in MCF-7(WT) cells (*p* < 0.01). Compared with the WT MCF-7 cells, phosphorylated proteins PI3K, Akt, and mTOR were significantly increased by 2.11-, 2.20- and 2.11-fold, respectively. By contrast, the total amount of these proteins did not change significantly, which implied that the up-regulation of Survivin in MCF-7/Survivin cells could activate the PI3K/Akt/mTOR signaling pathway by promoting the phosphorylation of these proteins. Recent studies found that the *MDR1* gene was a downstream gene regulated by mTOR phosphorylation (Wang L. et al., 2016). Western blotting analysis also showed that the expression of P-gp in MCF-7/Survivin cells was significantly up-regulated (2.22-fold increase). After further lowering the Survivin expression in MCF-7/Survivin cells by doxycycline, the phosphorylation of PI3K, Akt, and mTOR in the MCF-7/Survivin cells was significantly reduced, and P-gp expression was significantly down-regulated as well. After treatment with 2,250 nmol/L doxycycline for 24 h, the expression of Survivin in the cells decreased by 10.80-fold, and the expression of phosphorylated proteins (p-PI3K, p-Akt, p-mTOR) and P-gp decreased by 10.98, 9.05, 4.61 and 10.85-fold, respectively. However, the total amount of proteins PI3K, Akt, and mTOR still did not change significantly **(** Figure 6B **)**.

## Discussion

### High Expression of Survivin and P-Gp Are Related to the Occurrence of MDR

The occurrence of chemotherapy failure in cancer patients is usually related to MDR progression (Adamska et al., 2018). The underlying mechanism of the emergence of MDR is rather complicated (Szakács et al., 2006; Martin et al., 2014; Norouzi-Barough et al., 2018). Studies showed that the membrane transporter was the leading player responsible for pumping out intracellular drugs or gathering drugs from target sites to lysosomes to reduce drugs’ toxicity to cells (Pan et al., 2016; Adamska et al., 2018; Norouzi-Barough et al., 2018). P-gp is a two-domain membrane transport protein with nucleotide-binding activity. It plays an essential role in the cell’s resistance to various natural products (Eckford and Sharom, 2009; Liu et al., 2014; Wang C. et al., 2016; Dong et al., 2020), such as anthracyclines, vinca alkaloids, actinomycins, and violet taxanes (Han et al., 2016; Kawami et al., 2018; Guo et al., 2019). In addition to these membrane transporters, IAPs proteins are also an essential player in mediating drug resistance (Liu et al., 2007). As a member of IAPs, Survivin has received more and more attention because of its anti-apoptotic effects and tumor chemotherapy resistance (Wang et al., 2013; Rivadeneira et al., 2015; Garg et al., 2016; Khan et al., 2017). In terms of anti-apoptosis, Survivin does not seem to bind directly to caspase. On the contrary, Survivin plays an anti-apoptotic control effect by binding and stabilizing XIAP, which inhibits caspase-9 (McKenzie and Grossman, 2012). And several studies had shown that the expression of Survivin in drug-resistant tumor cells was significantly higher than that of normal cells (Lamers et al., 2012), and inhibition of Survivin could increase the sensitivity of resistant cells to chemotherapy drugs (Lamers et al., 2012). Our previous study showed that a dominant-negative mutant of Survivin, TATm-Survivin (T34A), increased the sensitivity of MCF-7/DOX cells to DOX (Xu et al., 2012). These findings proved that Survivin was involved in the MDR of tumors. This study found that MCF-7/DOX cells were highly resistant to DOX, 5-FU, and paclitaxel. The mRNA and protein levels of Survivin and P-gp in MCF-7/DOX cells were also significantly higher than that in the normal cells, which indicated that the drug resistance of MCF-7/DOX cells was closely related to the high expression of Survivin and P-gp. Several anticancer drugs (e.g., Pantoprazole (Liu et al., 2017), Tanshinone-1 (Xu et al., 2013), Resveratrol (Wang L. et al., 2016), Ubenimex (Guo et al., 2019)) could down-regulate the expression of P-gp through the PI3K/Akt/mTOR pathway, thereby enhancing the anticancer effect of chemotherapy drugs (Liu et al., 2014; Han et al., 2016; Chi et al., 2017). Similarly, our results confirmed that Survivin might affect the PI3K/Akt/mTOR pathway. Studies showed that the expression levels of Survivin and P-gp in MDR cell lines were higher (Liu et al., 2010), but the connection between the two proteins remains elusive.

### The Synergistic of Survivin With P-Gp Might Mediate MDR

CRISPR/Cas9 technology can precisely target the target gene and is simple to operate. It only needs to transfect a plasmid to accurately edit the target gene (Nakade et al., 2014). The Tet-off system can strictly control the expression level of the target gene according to the amount of exogenous doxycycline added, thereby achieving the purpose of real-time regulation (Mizuguchi and Hayakawa, 2002). The CRISPR/Cas9 plasmid is co-transfected with the target fragment containing the Tet-off system, and the corresponding fragment can be inserted at the designated position. It is not only easy to operate, but also precise targeting and regulation. In this present study, CRISPR/Cas9 technology and Tet-off regulatory system were used to successfully establish a quantitatively regulated system to control the expression of Survivin in the breast cancer cell MCF-7 (when the concentration of doxycycline was ≤6,750 nmol/ml, it will not affect the viability of MCF-7 cells within 72 h). The expression of Survivin in MCF-7/Survivin cells was in inverse proportion to the concentration of inducer doxycycline. Our results showed that Survivin overexpressed in MCF-7/Survivin cells could enhance cells’ resistance to apoptosis induced by DOX (drug resistance index was determined as 21.96). However, doxycycline could reverse the resistance of MCF-7/Survivin cells to DOX. Under 2,250 nmol/L doxycycline, the reversal index of MCF-7/Survivin cells to DOX was as high as 118.07. DOX is one of the substrates that P-gp can transport. When doxycycline was added, the DOX concentration in MCF-7/Survivin cells gradually increased because of weakened transport of DOX out of the cells caused by gradually decreased expression of P-gp. Calcium channel blocker verapamil could selectively inhibit the efflux function of P-gp (favoring the accumulation of DOX inside the cells) (Tsubaki et al., 2014; Peng et al., 2020), but did not affect the cellular level of P-gp. We used verapamil to create the situation in which only the function of P-gp was specifically inhibited. Unlike the verapamil’s function, down-regulated Survivin expression in MCF-7/Survivin cells inhibited P-gp and efflux function, leading to the reversal of anti-apoptosis and drug resistance to DOX. This phenomenon was similar to the results of Liu *et al.*(Liu et al., 2007). Another study reported that Survivin overexpressed in the human epidermoid carcinoma cells KBv200 and breast cancer cells MCF-7/Adr did not directly interact with P-gp (Shi et al., 2007), which implied that Survivin might affect the expression and transport activity of P-gp in cancerous cells via other mediator molecules. Actually, this new system can be used not only to clarify the Survivin-P-gp axis, but also explain the relationship between other related genes.

### Survivin Might Mediate Tumor Cell’s MDR via the PI3K/Akt/mTOR Pathway

PI3K/Akt/mTOR signaling pathway was necessary for tumor cells’ survival (Khan et al., 2013; Riquelme et al., 2016; Chen et al., 2018; Liu et al., 2020; Chen et al., 2021) (e.g., breast cancer (Yin et al., 2017; Nunnery and Mayer, 2020), head and neck cancer (Marquard and Jücker, 2020), and lung cancer (Tan, 2020)). This pathway also played an essential role in promoting cell growth, proliferation, metastasis, and chemotherapy resistance (Khan et al., 2013; Ahmad et al., 2020; Liu et al., 2020). It was reported that the P-gp-encoding gene *MDR1* was regulated by mTOR (Liu et al., 2017). Blocking PI3K/Akt/mTOR pathway with the specific inhibitor (LY294002) could reduce the basal level of P-gp and antagonize multi-drug resistance (Wang C. et al., 2016). Meanwhile, overexpression of Survivin enhanced the phosphorylation of PI3K and induced the phosphorylation of PI3K’s downstream factors Akt and mTOR (Ou et al., 2014; Sandra and Khosravi-Far, 2014; Sandra, 2018). When L929 cells were administered with inactive mutant Survivin (Ser81Ala), hypophosphorylation of Akt and mTOR was observed (Sandra, 2018). Also, Sandra indicated that Ser81 Survivin could play an important role in inducing PKA/PI3K/Akt/mTOR survival signaling pathway. A series of experiments proved that Survivin induced Akt phosphorylation via PI3K, and induced mTOR phosphorylation via Akt (Sandra, 2018). And McKenzie *et al.*(McKenzie et al., 2010) indicated that Survivin overexpression activated the Akt and mitogen-activated protein kinase pathways in melanocytes. Based on these observations, we deduced that Survivin might regulate the expression of P-gp through the PI3K/Akt/mTOR pathway, thereby playing a role in the chemotherapy resistance of tumor cells. Therefore, the expression of component proteins in the PI3K/Akt/mTOR pathway was detected. Western blotting results showed that the up-regulation of Survivin in MCF-7/Survivin cells could affect the PI3K/Akt/mTOR signaling pathway and increase the phosphorylation of PI3K, Akt, and mTOR. After further down-regulation of Survivin expression in MCF-7/Survivin cells by doxycycline, the phosphorylation of PI3K, Akt, and mTOR in the MCF-7/Survivin cells was significantly reduced, and the expression of P-gp was also significantly down-regulated. This is because Survivin as an upstream protein, its changes will cause many protein changes, not only phosphorylation-PI3K/Akt/mTOR and P-gp, but also may affect the expression of other proteins, and ultimately these proteins may affect the expression levels of phosphorylation-PI3K/Akt/mTOR and P-gp. Such a cascade reaction can cause the expression levels of phosphorylated-PI3K/Akt/mTOR and P-gp vary more. Therefore, the down-regulation of Survivin protein may lead to greater down-regulation of phosphorylated-PI3K/Akt/mTOR and P-gp. Studies showed that transcription of Survivin’s encoding gene *BIRC5* was associated with P-gp overexpression in the MDR MCF-7 cells (Reis et al., 2011), and Survivin expressed in drug-resistant cells did not directly interact with P-gp (Shi et al., 2007). In accord with these previous observations, our study found that Survivin might affect P-gp expression by affecting the PI3K/Akt/mTOR pathway, thereby mediating the emergence of MDR.

Using the MCF-7/Survivin cell line created with the CRISPR/Cas9 genome editing technology, we proved that Survivin might mediate tumor cell’s MDR via the PI3K/Akt/mTOR pathway. Additionally, we found that quantitatively inhibiting the expression of Survivin could sensitize the chemoresistant cell to the apoptosis-inducing drugs. We believe that our results will deepen the understanding of MDR of tumor cells and inspire the methods to improve chemotherapy’s efficiency by reversing the MDR.

## Data Availability

The datasets presented in this study can be found in online repositories. The names of the repository/repositories and accession number(s) can be found below: https://www.oncomine.org/resource/main.html, oncomine.
