# Supplementary material for: The Establishment of Quantitatively Regulating Expression Cassette with sgRNA Targeting BIRC5 to Elucidate the Synergistic Pathway of Survivin with P-Glycoprotein in Cancer Multi-Drug Resistance
Source: Front Cell Dev Biol. 2022 Jan 3;9:797005. doi: 10.3389/fcell.2021.797005 (PMC8762277; doi:10.3389/fcell.2021.797005)
Supplement: Supplementary file 1 [file DataSheet1.pdf]

The Establishment of Quantitatively Regulating Expression Cassette with sgRNA Targeting *BIRC5* to Elucidate the Synergistic Pathway of Survivin with P-Glycoprotein in Cancer Multi-Drug Resistance

Running title: How Survivin reverses cancer chemoresistance

Changping Deng[1], Fabiao Hu[1], Zhangting Zhao[1], Yiwen Zhou[1], Yuping Liu[2], Tong Zhang[2], Shihui Li[1], Wenyun Zheng[2†], Wenliang Zhang[3], Tianwen Wang[4†], Xingyuan Ma[1†]

[1]*State Key Laboratory of Bioreactor Engineering, East China University of Science and Technology, Shanghai 200237, P. R. China*
[2]*Shanghai Key Laboratory of New Drug Design, School of Pharmacy, East China University of Science and Technology, Shanghai 200237, P. R. China*
[3]*Center of Translational Biomedical Research, University of North Carolina at Greensboro, Greensboro, North Carolina 27310, United States*
[4]*College of Life Sciences, Xinyang Normal University, Xinyang 464000, P. R. China*

[†]*Correspondence:*
Xingyuan Ma
maxy@ecust.edu.cn
Tianwen Wang
tianwenw@gmail.com
Wenyun Zheng
zwy@ecust.edu.edu.cn

Supporting Information

Figure S1. Selection of sgRNA and construction of vector.

Figure S2. Detection of transfection efficiency of different sgRNA.

Figure S3. Schematic diagram of T7E1 digestion test and its primer design.

Figure S4. Expression cassette construction process.

Figure S5. Tet-off system regulated the expression of EGFP in MCF-7 cells.

Figure S6. Identification of positive cells.

Table S1. Potential off-target sites for sgRNA targeting *BIRC* loci.

Table S2. Primers used in this study.

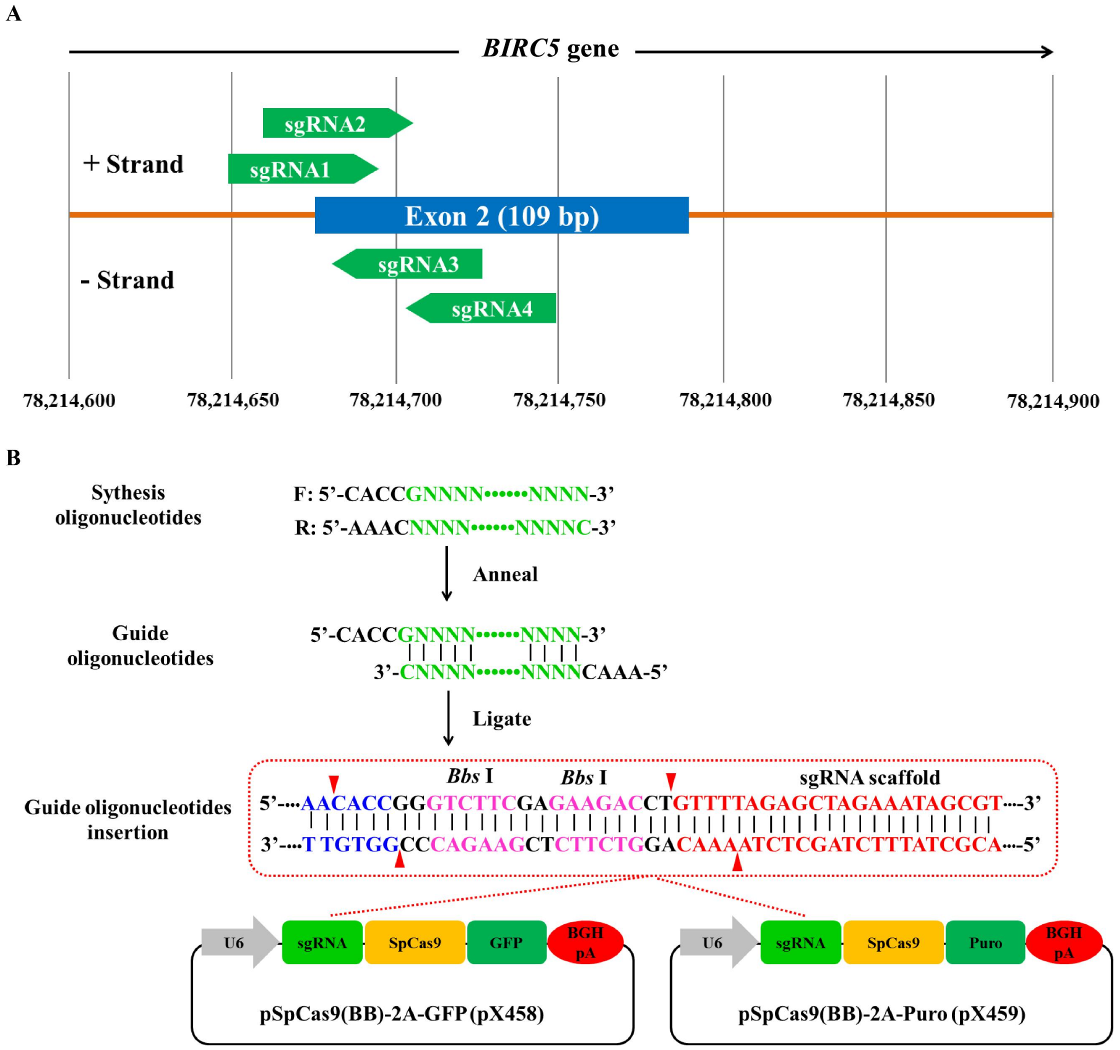

Figure S1. Selection of sgRNA and construction of vector. (A) Design position of sgRNA. (B) pX458 and pX459 vectors construction process.

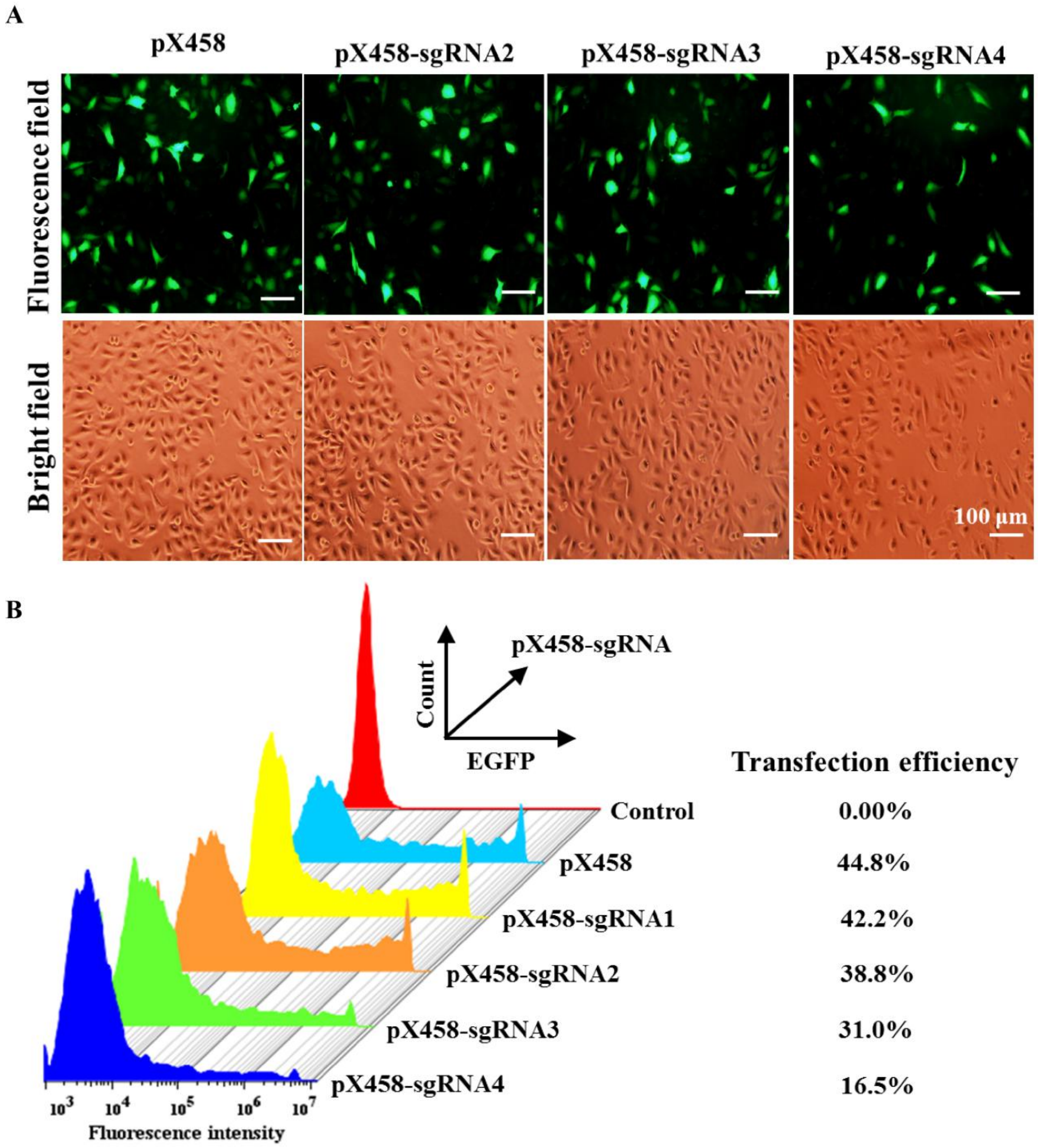

Figure S2. Detection of transfection efficiency of different sgRNA. (A) Fluorescence images and (B) transfection efficiency of MCF-7 cells transfected with different pX458-sgRNA plasmids. All images were taken at 100 × magnification (Bar = 100 μm).

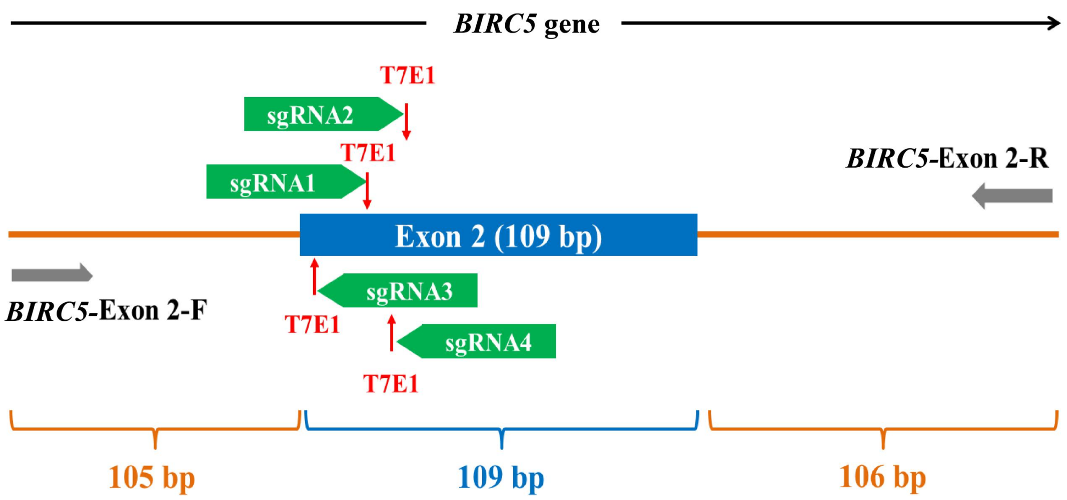

***BIRC5*-Exon 2-F: AGGCCGGCCTCCCCTCCCTGCTTTG**
***BIRC5*-Exon 2-R: ATGAGGGTGGAAAGCAACCCTCCCA**

Figure S3. Schematic diagram of T7E1 digestion test and its primer design.

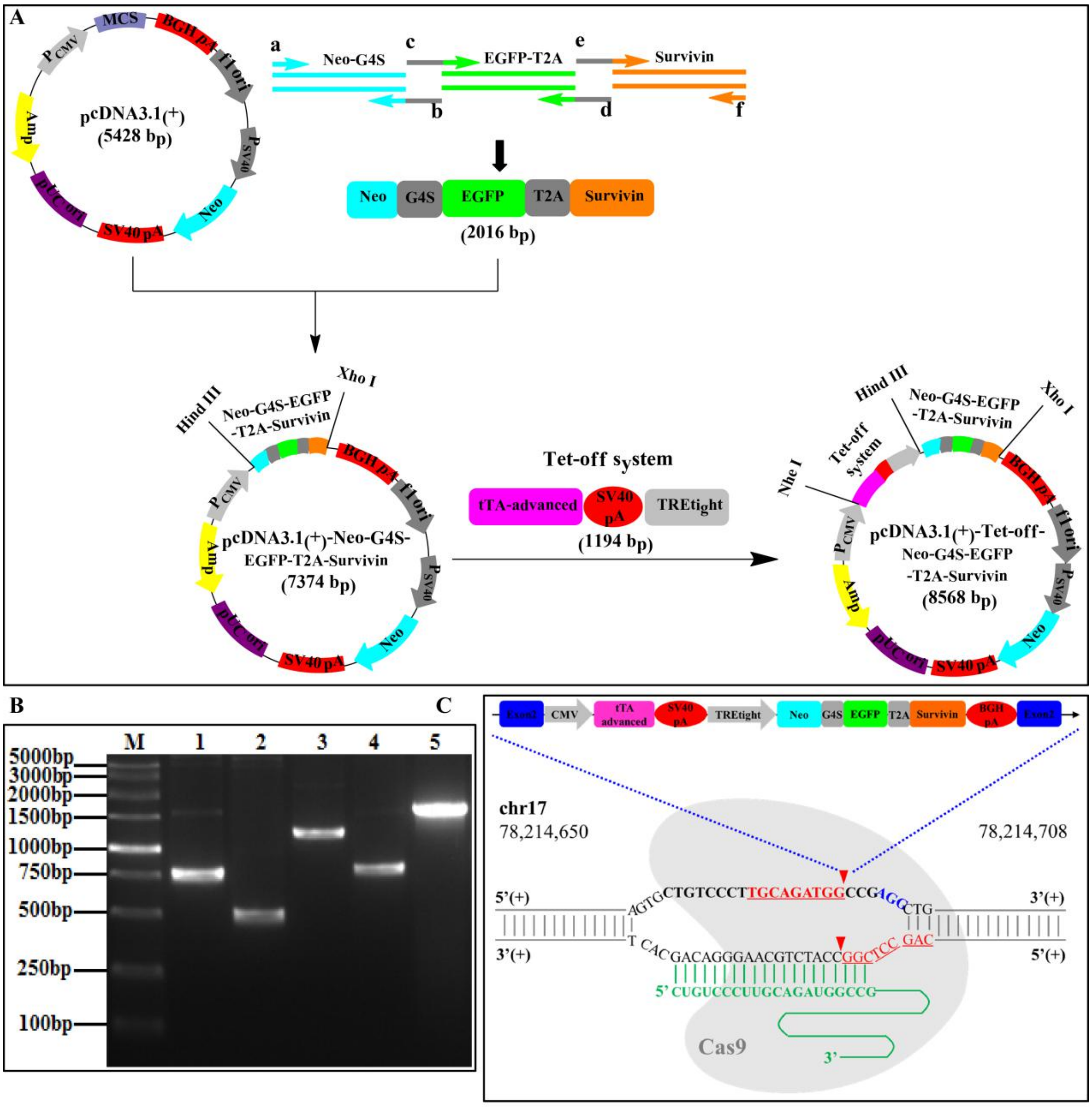

Forward primer: TGCAGATGGGACATTGATTATTGACTAGTT
Reverse primer: CAGCCTCGGCCATAGAGCCCACCGCATCCC

Figure S4. Expression cassette construction process. (A) Schematic diagram of the construction of linear expression cassette. (B) PCR amplification of different gene elements. Lane M, DL5000 Marker; lane 1, EGFP-T2A; lane 2, Survivin; lane 3, EGFP-T2A-Survivin; lane 4, Neo-G4S; lane 5, NETS. (C) Schematic diagram of the site-directed integration of linear expression cassette into *BIRC5* gene and its micro-homologous primer.

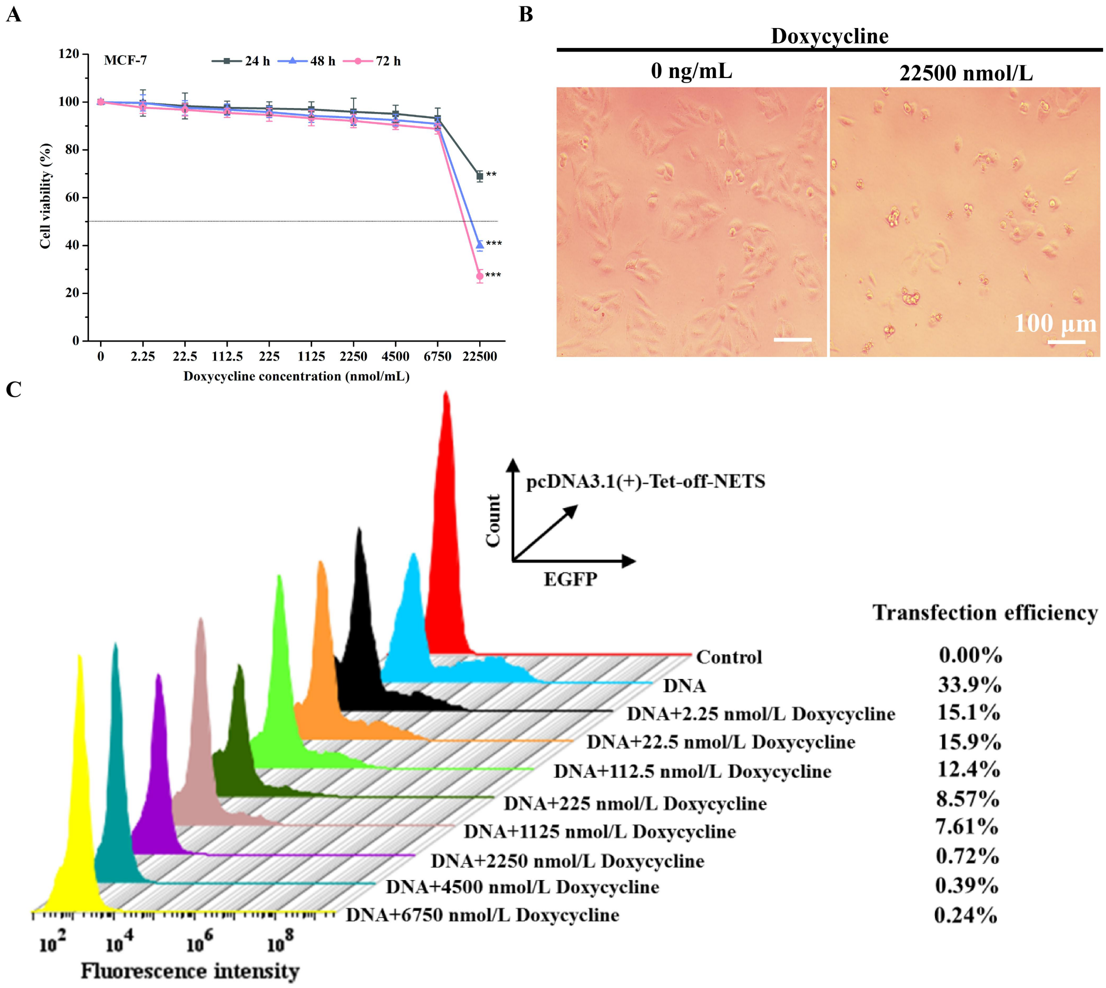

Figure S5. Tet-off system regulated the expression of EGFP in MCF-7 cells. (A) Cell viability of MCF-7 cells incubated with doxycycline for 24, 48, and 72 h, respectively. (B) The cell morphological changes after treated with doxycycline for 72 h. (C) Transfection efficiency of MCF-7 cells treated with pcDNA3.1(+)-Tet-off-NETS and different concentrations of doxycycline for 24 h. All images were taken at 100 × magnification (Bar = 100 μm). Data were expressed as mean ± SD (n = 3). $^{**}P < 0.01$ and $^{***}P < 0.001$.

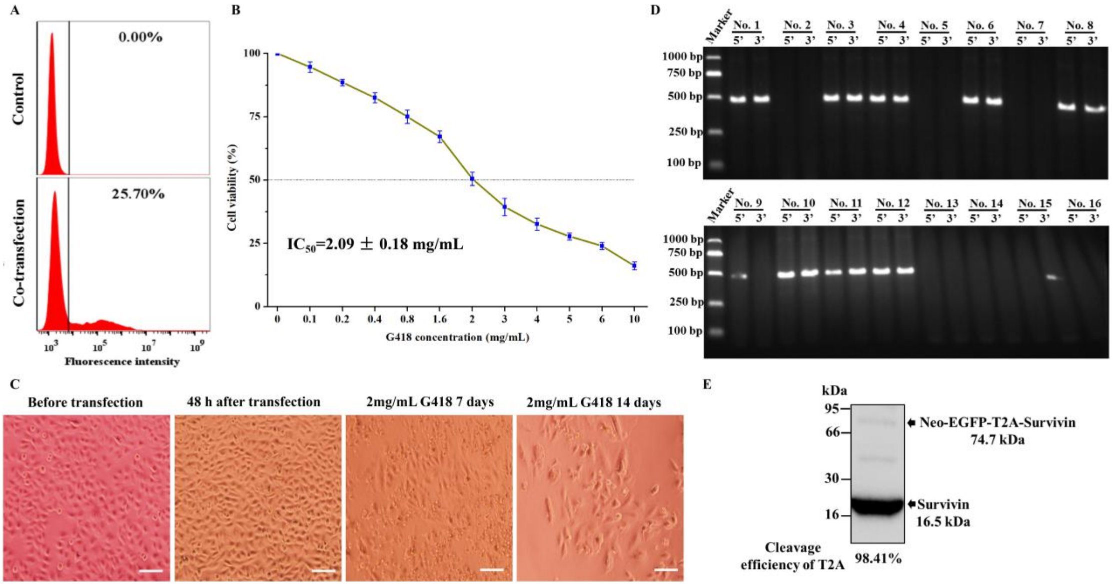

Figure S6. Identification of positive cells. (A) Co-transfection efficiency of pX459-sgRNA1 and linear expression cassette in MCF-7 cells. (B) Cell viability and IC_{50} of MCF-7 cells incubated with G418 for 48 h. (C) The morphological changes of MCF-7 cells treated with 2 mg/mL G418. All images were taken at 100× magnification (Bar = 100 μm). (D) Junction PCR identification of positive monoclonal cell lines. (E) The cleavage efficiency of T2A in Neo-EGFP-T2A-Survivin monitored by western blotting. Data were expressed as mean ± SD (n = 3).

Table S1. Potential off-target sites for sgRNA targeting *BIRC* loci.

ID	Off-target site (5'-3')	Score	Mismatch	Chromosome: Cut site	PAM	Target gene
Off-target sites of sgRNA1						
sgRNA1	CTGTCCCTTGCAGATGGCCG	100	0	chr17:78,214,650	AGG	*BIRC5* (Exon 2)
1	CTGTCCCCTGCAGGAGGCCG	0.9	3	chr21:45,934,739	GGG	*PCBP3*
2	CTGTCCCTTGCAGATGGTGA	0.8	3	chr8:100,988,071	TGG	--
3	CTGTCCCTTGCAGATGCTGG	0.7	3	chr20:62,624,131	TGG	--
4	CTGTCCCTAGCAGGTGGCTG	0.7	3	chr16:84,052,221	CGG	--
5	CTGACCCTTGCTGATGGCAG	0.6	3	chr10:12,842,502	GGG	--
6	CTGTTCATTGCAGATGGCAG	0.5	3	chr6:115,205,016	GGG	--
7	CTTTCCCTTGCAAATGACCG	0.4	3	chr2:207,863,037	GGG	*PLEKHM3*
Off-target sites of sgRNA2						
sgRNA2	CCCTTGCAGATGGCCGAGGC	100	0	chr17:78,214,654	TGG	*BIRC5* (Exon 2)
1	CCCATGCAGATGGCCAAGAC	1.3	3	chr22:26,126,286	TGG	--
2	CCCTTGCAGATGGCAGACTC	1.2	3	chr16:58,509,162	TGG	*NDRG4*
3	CCCTTCCAGATGGCCCAGCC	1.1	3	chr5:177,358,257	GGG	*RGS14*
4	TCCTTGCAGAGGGCCTAGGC	0.9	3	chr15:51,814,122	TGG	*TMOD2*
5	CCCTTGCAGATGGCCCTGGA	0.7	3	chr16:88,767,253	TGG	*PIEZO1*
6	CCCTTGCAGATGGACAAAGC	0.6	3	chr7:141,463,421	CGG	*TMEM178B*
Off-target sites of sgRNA3						
sgRNA3	GCAGTGGATGAAGCCAGCCT	100	0	chr17:78,214,666	GGG	*BIRC5* (Exon 2)
1	GCAGAGGATGAAGCCAACCT	1.1	2	chr10:107,137,214	TGG	*SORCS1*

2	GAAGTGGATGGAGCCAGCCT	1.0	2	chr4:26,125,340	GGG	--
3	GCAGTGGATGAAGCCTCCAT	0.8	3	chr5:179,340,223	GGG	*ADAMTS2*
4	ACAGTGCATGTAGCCAGCCT	0.7	3	chr6:25,678,766	GGG	*SCGN*
5	GCAGGTGATGAAGCCAGCCA	0.5	3	chr12:29,840,190	GGG	--
Off-target sites of sgRNA4						
sgRNA4	GCTCGTTCTCAGTGGGGCAG	100	0	chr17:78,214,682	TGG	*BIRC5* (Exon 2)
1	GCTCGTTCCAAGTGGGGCTG	1.5	3	chr2:134,278,736	AGG	*MGAT5*
2	ACTCGGTCTCAGTGGGGCAA	1.4	3	chr7:102,859,678	AGG	*FBXL13*
3	GCTTGAACTCAGTGGGGCAG	1.2	3	chr9:127,377,719	AGG	*GARNL3*
4	GATCTTTCTGAGTGGGGCAG	1.1	3	chr20:57,428,925	GGG	--
5	GCTCTTTGTGAGTGGGGCAG	1.0	3	chr22:17,007,931	TGG	*AC006548.28*
6	GCTCGGTCTCAGAGGGTCAG	0.9	3	chr14:101,635,078	TGG	*RP11-1029J19.5*
7	GCTGGTGCTCAGTGGGTCAG	0.8	3	chr2:233,795,568	TGG	*MROH2A*
8	GCTGGTTCCCAGTGGGTCAG	0.6	3	chr17:73,595,696	CGG	*SDK2*

Note: The underline was the mismatched base.

Table S2. Primers used in this study.

Named	Primer sequence (5’→3’)		Used
sgRNA1	F: CACCGCTGTCCCTTGCAGATGGCCG		Designed-sgRNA
	R:AAACCGGCCATCTGCAAGGGACAG		
sgRNA2	F: CACCGCCCTTGCAGATGGCCGAGGC		
	R:AAACGCCTCGGCCATCTGCAAGGG		
sgRNA3	F: CACCGAGGCTGGCTTCATCCACTGC		
	R:AAACGCAGTGGATGAAGCCAGCCT		
sgRNA4	F: CACCGCTGCCCCACTGAGAACGAGC		
	R:AAACGCTCGTTCTCAGTGGGGCAG		
Neo	F:CCCAAGCTTGCCACCATGATTGAACAAGATGGATTG	*Hind* III Kozak	Constru-ction of linear expressio-n cassette
	R:AGAACCTCCTCCACCGAAGAACTCGTCAAGAAGGCG	G4S	
EGFP	F:GGTGGAGGAGGTTCTATGGTGAGCAAGGGCGAGGAG	G4S	
	R:GGCATGGACGAGCTGTACAAGGGCAGTGCAGAGGGCAGAGGAAGTCTGCTAACATGCGGTGACGTCGAGGAGAATCCTGGCCCA	T2A	
Survivin	F:GGCAGTGCAGAGGGCAGAGGAAGTCTGCTAACATGCGGTGACGTCGAGGAGAATCCTGGCCCAATGGGTGCCCCGACGTTGCCC	T2A	
	R: CCGCTCGAGTCAATCCATGGCAGCCAGCTG	*Xho* I	
5’ F_{out}	F:CCCAGAAGGCCGCGGGGGGT		Junction PCR
5’ R_{in}	R:ACTAGTCAATAATCAATGTC		
3’ F_{in}	F:GATGCGGTGGGCTCTATGG		
3’ R_{out}	R:TTCAAAGGGTAATTTTTGTG		
β-actin	F:ATTGGCAATGAGCGGTTC		qRT-PCR
	R:GGATGCCACAGGACTCCAT		
Survivin	F:AAGAACTGGCCCTTCTTGGA		
	R:CAACCGGACGAATGCTTTT		
P-gp	F:GGCTCCGATACATGGTTTTCC		
	R:TTCAGTGTGCGATCTTCCCAGC		
